# Impact of anterior commissure invasion on the oncological efficacy of transoral laser microsurgery for T1 glottic cancer

**DOI:** 10.3389/fonc.2025.1584313

**Published:** 2025-09-04

**Authors:** Qi Wang, YangYiYi Huang, Yun Li, Yi Ling, JianSheng Zhou, ZheWei Lou, Guo-Kang Fan

**Affiliations:** Department of Otolaryngology, The Second Affiliated Hospital Zhejiang University School of Medicine, Hangzhou, China

**Keywords:** glottic cancer, larynx, anterior commissure, transoral laser microsurgery, survival analysis

## Abstract

**Background:**

In glottic laryngeal cancer, anterior commissure (AC) involvement affects prognosis. T staging ignores AC subregion involvement and TNM staging may not suit as an AC prognostic indicator. We explored how AC involvement degree impacts prognosis in T1 glottic laryngeal cancer treated with transoral laser microsurgery (TLM). Methods: A retrospective study included 367 T1 glottic cancer patients undergoing TLM in the Department of Otorhinolaryngology at the Second Affiliated Hospital of Zhejiang University School of Medicine from January 2012 to June 2024. There were 348 males and 19 females, aged 42 – 87 years (average 63.7, SD 8.9). The median follow-up was 49.5 months (6.2 - 190.7). Staging was performed according to the degree of AC involvement (AC0–AC3) as suggested by Rucci L, and we analyzed its impact on prognosis.

**Results:**

No major complications occurred. Twenty-five patients had recurrence (6 open surgery, 17 repeat TLM, 2 palliative treatment); 4 died. The 5-year local control rate for T1 was 91.5%, and the disease-related survival rate was 98.3%. Specifically, for T1a, they were 92.3% and 99.0% respectively, and for T1b, they were 87.5% and 97.6% respectively. Patients with severe AC involvement (AC3) significantly affected the local control rate (p = 0.014), and the 5-year local control rate for AC3 was 61.0%. Intraoperative margin pathological examination had an impact on the local control rate and the disease-related survival rate (the p values of the log-rank test were 0.011 and 0.045 respectively). Poorly differentiated pathological results had an impact on the disease-related survival rate (the p value of the log-rank test was 0.001). Univariate analysis of the Cox proportional hazards regression model showed that margin pathological examination and AC3 were important negative prognostic factors for the local control rate.

**Conclusion:**

TLM shows good oncological efficacy in the treatment of T1 glottic cancer. Our study provides evidence that it is not the T stage but the severe involvement of the AC that affects the prognosis of T1a and T1b. For patients with severe AC involvement, TLM should be chosen cautiously, and open partial laryngectomy may serve as an alternative option.

## Introduction

Laryngeal cancer is one of the most common malignant tumor types in the head and neck region. According to the data estimated by the International Agency for Research on Cancer (IARC) in 2022, it accounts for approximately 10% of all head and neck malignancies (1). The National Comprehensive Cancer Network (NCCN) of the United States points out that glottic cancer accounts for about 60 - 65% of the total laryngeal cancers (2). Early glottic cancer includes Tis, T1a, and T1b (3), and some scholars believe that it also includes the T2 stage (4). Tao et al. (5) reported that early glottic cancer accounted for 79% of all laryngeal cancer patients; in our hospital, the data was 80%, among which T1 accounted for 57%. T1 stage cancer is defined as a tumor that only involves the vocal cords and does not affect their mobility (6). The American Joint Committee on Cancer (AJCC) began to subdivide T1 stage glottic cancer into T1a and T1b stages in the third edition starting in 1988 (7). Because it was recognized that cancers involving one or both vocal cords had different prognoses and might require different treatment methods. However, anterior commissure (AC) is an important subregion of the larynx that has not been incorporated into the current T staging system.

Nowadays, due to the fact that Transoral Laser Microsurgery (TLM) can preserve organs and functions, has a low incidence of aspiration, and a short hospital stay, it has become a widely accepted treatment method for T1 glottic cancer, which has gradually reduced the number of open laryngectomies. However, there are still many controversies and uncertainties regarding issues such as AC involvement, margin analysis, staged surgeries, and simultaneous wound repair.

This study analyzed the oncological efficacy of TLM for T1 glottic cancer and explored the impact of common risk factors and the degree of AC involvement on the prognosis.

## Materials and methods

This retrospective study was conducted on 367 patients in the Department of Otorhinolaryngology, the Second Affiliated Hospital of Zhejiang University School of Medicine. The data were sourced from the medical records of patients with laryngeal squamous cell carcinoma who received TLM treatment from January 1, 2009 to June 30, 2024. Inclusion criteria: The cancer was primary and diagnosed as squamous cell carcinoma pathologically. It was glottic laryngeal cancer with the stage of T1N0M0. CT evaluation was performed to exclude spread to the paraglottic and cartilage invasion. Radiological evidence of erosion of the inner cortex of the thyroid cartilage, and involvement of the paraglottic space, patients were referred to other treatment options. Patients with poor exposure of the anterior commissure were also not considered suitable for TLM. No radiotherapy had been carried out after the first operation. Neither chemotherapy for other tumors nor radiotherapy for the head and neck region had been conducted previously. This study was approved by the Institutional Ethics Committee (approval number No. 2024 - 1545). There were a total of 367 patients, including 348 males and 19 females, aged between 42 and 87 years (with an average age of 63.7 (SD 8.9) years). From the date of surgery to December 31, 2024, outpatient follow-up visits and telephone interviews were used to follow up with the patients themselves or their family members, and the median follow-up time was 49.5 (ranging from 6.2 to 190.7) months.

The diagnosis and TNM staging of laryngeal cancer were determined by electronic laryngoscopy, neck and abdominal ultrasound, chest and neck CT, and laryngeal biopsy and histopathological examination. This study included patients diagnosed with glottic cancer (T1N0M0) and with no previous history of treatment for laryngeal malignancies. The presence or absence of AC involvement and the classification of vocal cord resection types were carried out according to the surgical records in our hospital’s EMRS case system and previous electronic laryngoscopy photos.

**Figure 1:**
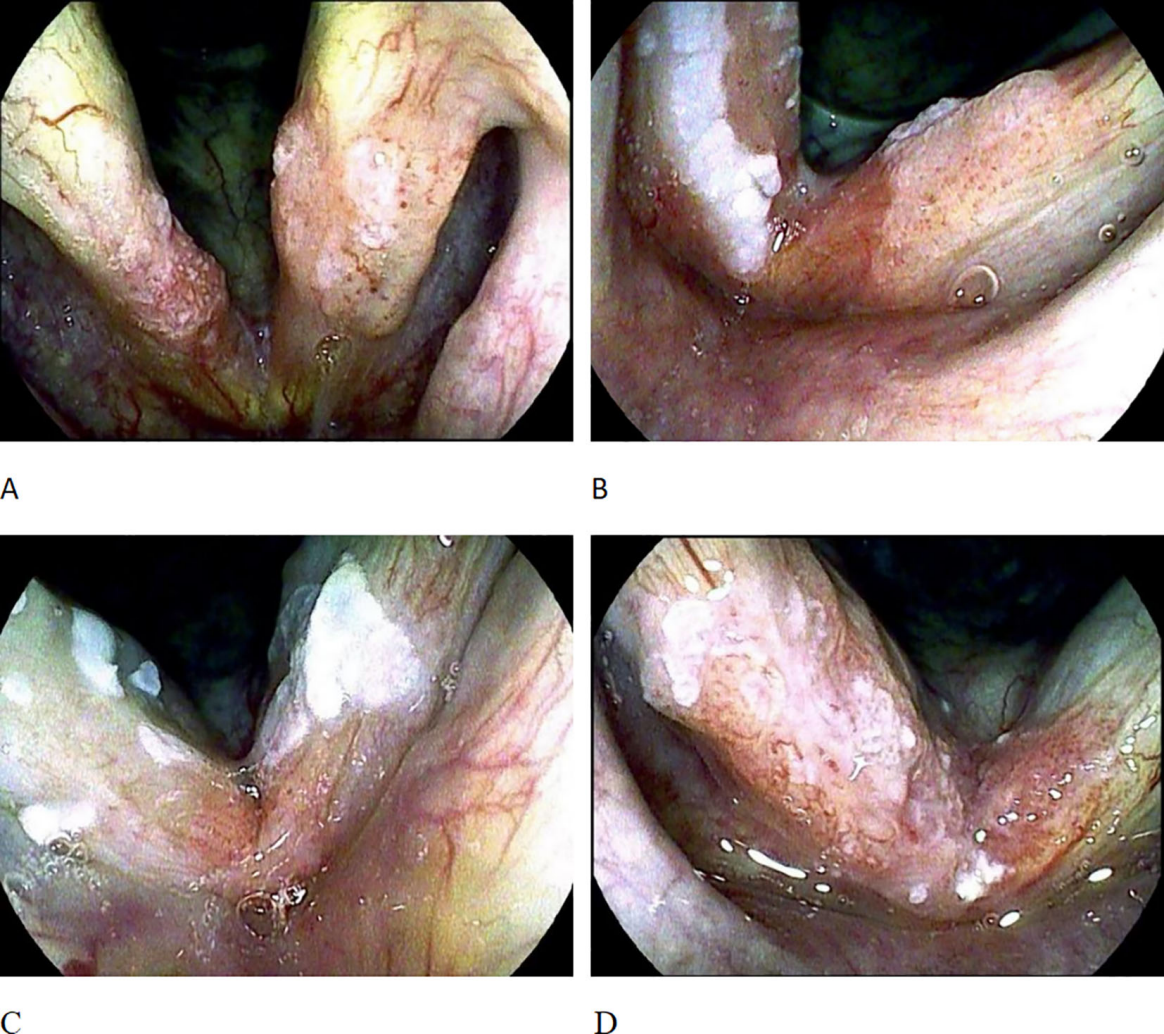
AC staging. **(A)** shows AC0, with bilateral vocal cord lesions but the AC region not involved; **(B)** shows AC1, where the AC region is only involved on one side of the midline; **(C)** shows AC2, where the AC region is involved across the midline. **(D)** shows AC3, involvement of the whole anterior commissure subsite on both sides across the midline.

The TLM was performed under general endotracheal anesthesia. The old Sharplan 30C CO_2_ laser and the new AcuPulse30ST CO_2_ laser of Lumenis Corporation were successively used, with an output power of 3 - 8W in the super-pulse mode, and the operation was carried out under the Carl Zeiss surgical optical microscope. The tumor was resected along the safe margin (2 - 3mm) outside the edge of the tumor. For cases where preoperative pathological biopsy was not taken, tissue was first taken under the microscope for rapid frozen section pathological examination; for patients with pathologically confirmed laryngeal malignancies, the tumor was directly resected. The surgery was performed by a single surgeon using the “en bloc resection” or “piecemeal resection” technique according to the expansion and exposure of the tumor. The classification of vocal cord resection was based on the standards of the European Laryngological Society (ELS) (8, 9): type I, subepithelial; type II, subligamental; type III, transmuscular; type IV, total; type Va, encompassing the contralateral vocal fold and the AC; type Vb including the arytenoids; type Vc, encompassing the subglottis; type Vd, including the ventricle; and type VI, AC ectomy with bilateral anterior cordectomy. To prevent vocal cord adhesion, some patients received staged surgeries, laryngeal membrane implantation, and ventricular band mucosal flap repair. For T1a, Types II - Va vocal cord resection were adopted, and for T1b, Types II - VI vocal cord resection were used. Since 2016, a subset of patients have not undergone intraoperative frozen section analysis of surgical margins. Since 2019, the scope of the tumor was determined by preoperative examination with the image-enhanced endoscope i-scan of Pentax Corporation of Japan, and intraoperative frozen section analysis of surgical margins has not been performed. For patients with postoperative vocal cord adhesion, granulation residue, or partial local recurrence, secondary TLM treatment was performed. This study recorded the demographic characteristics (age and gender) of the patients, risk factors (smoking and drinking conditions), AC involvement, and postoperative vocal cord adhesion and granulation hyperplasia. Smoking habits were graded according to packs per year, and patients were divided into non-smokers or smokers with less than 20 packs per year (Light Smokers), smokers with 20 – 40 packs per year (Moderate Smokers), and smokers with more than 40 packs per year (Heavy Smokers). Ethanol exposure was graded according to the average daily ethanol intake, and patients were divided into those with no or minimal intake (Minimal Intake), those with mild to moderate intake (< 100 grams per day) (Moderate Intake), and those with heavy intake (> 100 grams per day) (Heavy Intake). All of the above factors were combined to determine the local control rate and disease-specific survival rate of the patients. The AC subarea classification referred to the classification proposed by Rucci et al. (10): AC0 indicated that both vocal cords were affected but the AC area was not involved; AC1 indicated that the AC area was only affected on one side of the midline; AC2 indicated that part of the AC area crossed the midline and was affected; AC3 indicated that the entire AC area was affected, and there were lesions on both the upper and lower surfaces of the AC (**Figure 1**).

Statistical analysis was performed using SPSS 25.0 software. Descriptive statistics were carried out on the demographic characteristics, risk factors, and other follow-up parameters, and they were presented in the form of frequencies and proportions. The Kaplan - Meier method and 95% confidence intervals were used to calculate the local control rate and the disease-specific survival rate, and the log-rank test was carried out. Univariate analysis was performed using the Cox proportional hazards regression model, and multivariate analysis was used to evaluate the impact of prognostic factors on the local control rate and the disease-specific survival rate. Statistical significance was set at p < 0.05.

## Results

**Figure 2:**
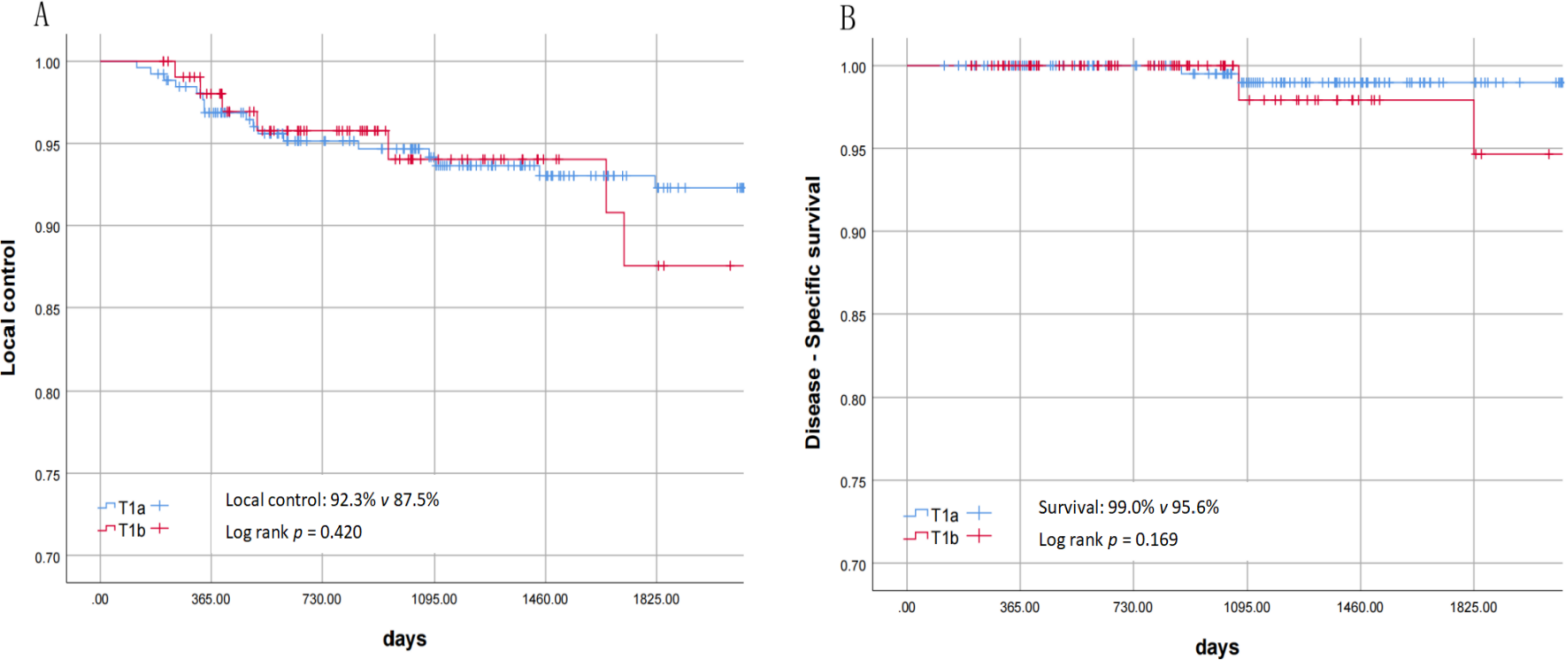
**(A)** Local control of T1a and T1b. **(B)** disease-specific survival rate of T1a and T1b.

The clinical data and statistical descriptions of 367 patients are shown in **Tables 1**, **2**. No major complications occurred in all patients (such as bleeding or edema requiring tracheotomy, airway deflagration, perichondritis, cartilage perforation, pneumonia, etc.). Six patients underwent corrective surgery due to severe vocal cord adhesion, and the surgical methods included direct laser resection and separation, laryngeal membrane implantation, or ventricular band mucosal flap repair of the wound. Ten patients had staged surgeries, and the interval between the second surgeries was 4 – 6 weeks. Sixteen patients underwent ventricular band mucosal flap to prevent or treat vocal cord adhesion. Recurrence occurred in 25 patients: 2 with isolated lymph node metastasis, 2 with local recurrence plus lymph node metastasis, 2 with local recurrence plus distant metastasis, and 19 with isolated local recurrence; treatment included open surgery (6 patients), repeat TLM (17 patients), and palliative therapy (2 patients). Four patients died: one patient was diagnosed as T4 at the time of recurrence and gave up treatment due to old age; one patient had recurrence combined with lung cancer and tongue cancer and chose non-surgical treatment; one patient died of pulmonary metastasis after recurrence; one patient with poorly differentiated pathology had no recurrence in the larynx but died due to multiple lymph node metastases in the neck and armpit. Calculated by the Kaplan - Meier method, the 3-year and 5-year local control rates of these 367 patients were 94.2% and 91.5% respectively (**Figure 2A**); the disease-related survival rates were 98.9% and 98.3% respectively (**Figure 2B**).

**Table 1:** Clinical characteristics and distribution of prognostic indicators in 367 patients undergoing transoral laser microsurgery.

Variable/Factor	No. of patients (%)	LC				DSS			
		Relapse	3-year (%)	5-Year (%)	Log rank p	Dead	3-year (%)	5-year (%)	Log rank p
Age					0.128				0.711
< 65	199 (54.2)	18	93.4	89.1		2	99.3	98.3	
≥ 65	168 (44.8)	7	95.1	95.1		2	98.4	98.4	
Gender					0.791				0.644
Male	348 (94.8)	24	94.2	91.3		4	98.8	98.2	
Female	19 (5.2)	1	94.4	94.4		0	100.0	100.0	
Smoking					0.918				0.384
Non/Light-smokers	54 (14.7)	4	93.9	90.1		0	100.0	100.0	
Heavy-Smokers	313 (85.3)	21	94.3	91.8		4	98.7	98.0	
Alcohol*					0.320				0.024
Non/Light-Consumers	152 (41.4)	8	95.7	93.4		0	100	100	
Moderate-Consumers	108 (29.4)	8	93.5	90.8		3	97.6	94.8	
Heavy-Consumers	107 (29.2)	9	92.7	89.2		1	98.7	98.7	
Intraop frozen margin*					0.011				0.045
Yes	121 (33.0)	5	96.7	95.8		0	100.0	100.0	
No	246 (67.0)	20	92.5	85.8		4	98.1	96.3	
Pathological grading*					0.180				0.02
well	322 (87.8)	19	95.1	92.7		3	99.2	98.5	0.001
moderately	39 (10.6)	5	88.5	84.1		0	100.0	100.0	0.023
poorly	6 (1.6)	1	83.3	83.3		1	83.3	83.3	
Type of cordectomy*					0.382				0.031
II	35 (9.5)	3	94.0	89.7		0	100	100	
III	153 (41.7)	10	93.2	91.1		1	99.0	99.0	
IV	63 (17.2)	3	96.6	93.8		0	100	100	
V	96 (26.1)	8	94.2	90.1		2	98.6	96.5	
VI	20 (5.5)	1	94.4	94.4		1	92.3	92.3	

LC, local control; DSS, disease-specific survival; AC, anterior commissure, *indicates statistically significant. Pathological grading*: Poorly differentiated vs. well-differentiated (p=0.001) and moderately differentiated (p=0.023).

Bold values indicate statistically significant differences.

**Table 2:** Impact of T staging and anterior commissure involvement degree on prognosis in patients treated with transoral laser microsurgery.

Variable/Stage	No. of patients (%)	LC				DSS			
		Relapse	3-year (%)	5-Year (%)	Log rank p	Dead	3-year (%)	5-year (%)	Log rank p
T staging					0.420				0.169
T1a	260	17	94.2	92.3		2	99.0	99.0	
T1b	107	8	94.0	87.5		2	98.9	95.6	
AC involvement					0.747				0.364
Yes	203 (55.3)	14	95.1	90.8		3	99.1	99.1	
No	164 (44.7)	11	93.1	92.0		1	98.8	97.5	
AC staging*					0.079				0.054
AC0	164	11	93.0	91.9	**0.024**	1	99.1	99.1	
AC1	138	8	95.4	93.0	**0.021**	1	99.1	99.1	
AC2	40	2	96.3	90.6	**0.014**	1	94.4	94.4	
AC3	25	4	91.5	61.0		1	94.1	94.1	

LC, local control; DSS, disease-specific survival; AC, anterior commissure; * indicates statistically significant. AC staging*: Significant differences between AC3 vs. AC0-AC2 (p=0.024, 0.021, 0.014).

Bold values indicate statistically significant differences.

**Figure 3:**
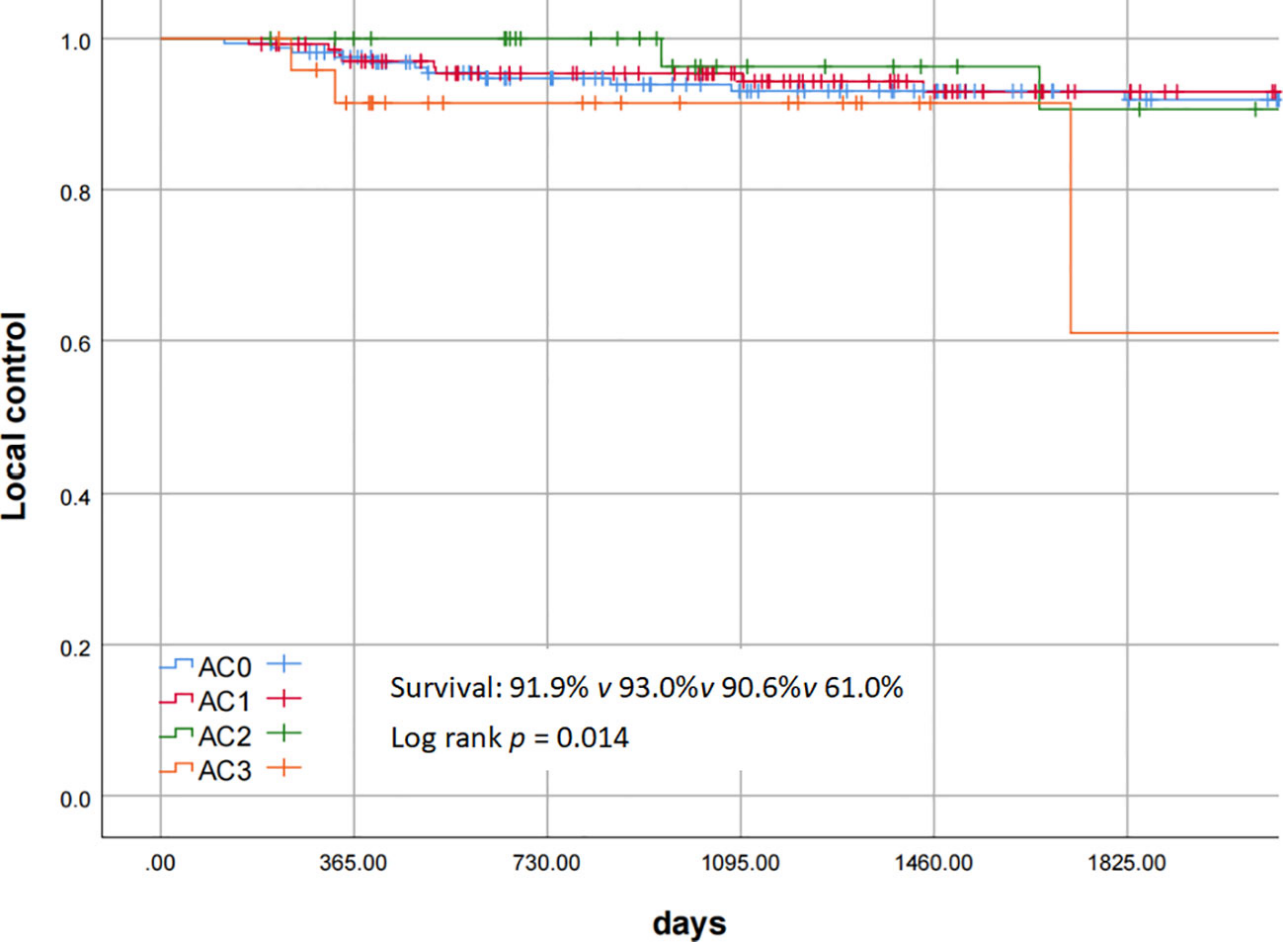
Local control of AC staging.

Postoperative pathological reports showed discrepancies in margin status assessment by different pathologists, with some lacking clear specification. Ten AC3 patients had negative margins at both ends and the base, compared to 15 with unspecified margins. Intraoperative margin pathological examination had an impact on the local control rate and the disease-related survival rate (the p values of the log-rank test were 0.011 and 0.045 respectively). Poorly differentiated pathological results had an impact on the disease-related survival rate (the p value of the log-rank test was 0.02). Patients with severe AC involvement (AC3) significantly affected the prognosis, and the 5-year local control rate for AC3 was 61.0% (**Figure 3**). Pairwise comparisons across differentiation grades revealed statistically significant differences between AC3 and AC0-AC2, with p-values of 0.024, 0.021, and 0.014 respectively. Univariate analysis of the Cox proportional hazards regression model is shown in **Tables 3**, **4**, which showed that margin pathological examination was an important negative prognostic factor for the local control rate (HR = 3.59 (95% CI: 1.28 - 10.08), p = 0.015). Multivariate analysis of the Cox proportional hazards regression model showed that margin pathological examination was an important negative prognostic factor for the local control rate (HR = 3.15 (95% CI: 1.06 - 9.39), p = 0.039).

**Table 3:** Cox univariate analysis of local control and disease-specific survival in 367 patients undergoing transoral laser microsurgery.

Variable	LC			DSS		
	HR	95% CI	p	HR	95% CI	p
Age						
≥ 65	1			1		
< 65	1.95	0.81-4.66	0.136	1.46	0.2.5-10.36	0.706
Gender						
Female	1			1		
Male	1.31	0.18-9.68	0.792	21.727	0.00-8.99E+9	0.761
Smoking						
Non/Light-smokers	1			1		
Heavy-Smokers	0.918	0.32-2.75	0.918	26.48	0.00-2.52E+6	0.575
Alcohol						
Non/Light-Consumers	1			1		
Moderate-Consumers	1.61	0.60-4.31	0.341	1.75E+5	0.00-6.02E+160	0.947
Heavy-Consumers	1.62	0.60-4.20	0.322	4.32E+4	0.00-1.62E+160	0.953
Intraop frozen margin*						
Yes	1			1		
No	3.59	1.28-10.08	**0.015**	78.55	0.23-2.66E+5	0.293
Pathological grading*						
well	1			1		
moderately	2.278	0.85-6.11	0.102	0.00	0.00	0.992
poorly	2.547	0.34-19.04	0.362	15.06	1.55-146.54	**0.019**
Type of laser cordectomy						
II	1			1		
III	0.858	0.23-3.12	0.816	2.39E+4	0.00-3.60E+240	0.731
IV	0.588	0.12-2.92	0.516	0.987	0.00-8.82E+307	1.000
V	1.05	0.28-3.97	0.942	6.74E+4	0.00-1.01E+241	0.968
VI	0.939	0.10-9.11	0.956	3.13E+5	0.00-4.73E+241	0.561
T staging						
T1a	1			1		
T1b	1.41	0.61-3.29	0.422	3.82	0.54-27.21	0.18

LC, local control; DSS, disease-specific survival; AC, anterior commissure, * indicates statistically significant.

Bold values indicate statistically significant differences.

**Table 4:** Cox univariate analysis of the impact of T staging and anterior commissure (AC) involvement degree on prognosis.

Variable	LC			DSS		
	HR	95% CI	p	HR	95% CI	p
T staging						
T1a	1			1		
T1b	1.41	0.61-3.29	0.422	3.82	0.54-27.21	0.18
AC involvement						
No	1			1		
Yes	1.14	0.515-2.52	0.747	2.73	0.28-26.38	0.384
AC staging*						
AC0	1			1		
AC1	0.92	0.37-2.30	0.862	1.24	0.08-19.89	0.879
AC2	0.77	0.17-3.49	0.739	4.52	0.28-72.47	0.286
AC3	3.55	1.11-11.31	**0.032**	12.40	0.75-204.87	**0.078**

LC, local control; DSS, disease-specific survival; AC, anterior commissure, * indicates statistically significant.

Bold values indicate statistically significant differences.

## Discussion

The oncological efficacies of TLM, radiotherapy, and open surgery are comparable in the treatment of T1 glottic cancer. Huang et al. (11) reported that among 818 patients with Tis and T1 glottic cancer treated by TLM, the 5-year overall survival rate was 92.6%, the disease-specific survival rate was 96.9%, and the local control rate was 91.3%. The 5-year local control rate of T1 glottic cancer with radiotherapy is 82 - 94% (12, 13), and that with open surgery is 91 - 100% (14, 15). Each treatment modality has its own limitations. Malik et al. (16) analyzed 22414 T1 patients in the National Cancer Database of the United States from 2004 to 2020. 57% of them only received radiotherapy, 21% only underwent surgery, and 22% received bimodal treatment (overtreatment). The proportion of surgeries has been increasing year by year. Lombardo et al. (17) compared and evaluated the voices of patients after TLM and radiotherapy for T1 glottic cancer. From an objective perspective, radiotherapy has an advantage; however, from the perspective of patients’ self-perception of voice quality, it cannot be considered that one treatment is more advantageous than the other. Van Loon et al. (18) found in a survey that 96% of patients with T1/2 glottic cancer preferred surgery, usually because they believed that choosing surgery first had advantages such as a shorter treatment time and the availability of subsequent treatment options if the surgery failed. Currently, the choice of treatment for T1 glottic cancer is largely influenced by institutional traditions, expert preferences, and equipment conditions. Ideally, the patient’s condition, the patient’s and their family’s understanding of the disease, their acceptance of different treatment modalities, and the patient’s need for organ function preservation should be comprehensively considered to select the most appropriate treatment plan.

Fang et al. (19) analyzed 5272 T1 patients in the Surveillance, Epidemiology, and End Results (SEER) database of the National Cancer Institute and the National Institutes of Health of the United States. After propensity score matching, there was no significantly better prognosis for T1a glottic cancer compared with T1b glottic cancer. Nomiya et al. (20) conducted a single-center study and reported that the 5-year disease-specific survival rate for T1 stage cancer was 95.9%, with 97.1% for T1a stage cancer and 93.4% for T1b stage cancer. The difference in survival outcomes between T1a and T1b glottic cancer may be related to AC involvement. Previous reports found that data from TLM, radiotherapy, and open surgery showed that patients with AC involvement had a relatively worse prognosis than those without involvement (21–23). On the other hand, in some studies on TLM and radiotherapy, no significant impact of AC involvement on the oncological prognosis was found (24, 25). The reason for the differences in views in previous retrospective studies may be the lack of analysis of the impact on the oncological prognosis from the perspective of the severity of AC involvement. Currently, the T staging does not take into account the involvement of the AC subarea, and the TNM staging may not be suitable as a prognostic indicator for the AC. In 1996, Rucci proposed the AC staging based on the degree of involvement of the AC in the horizontal and vertical planes. Carta et al. (26) used the AC staging to analyze T1 glottic cancer. The involvement of AC1 and AC2 had no statistically significant impact on the prognosis of patients, but the 5-year recurrence-free survival rate of 9 AC3 patients was 74.1% (p = 0.0446). There were no significant differences in the recurrence rate, overall survival rate, recurrence-free survival rate, and local control rate with laser alone between T1a and T1b tumors. Eker et al. (27) obtained results consistent with those of Carta. The 5-year local control rate of their 13 AC3 patients was 62.5%. They believed that AC3 was a significant adverse prognostic indicator after TLM, rather than the T stage. To our knowledge, our study is the largest cohort study on analyzing the impact of AC subarea classification on prognosis after TLM. Our study found that AC involvement had no significant negative impact on the overall local control rate of T1, but when the AC was severely involved (AC3, that is, the vertical plane of the AC was invaded), it would significantly affect the local control rate.

The desection of AC is a difficult area in the treatment of early glottic cancer. The scope of the AC includes: its front is located within the middle layer of the thyroid cartilage, its rear is the frontal plane passing through the rear of the anterior quarter of the vocal cord; its upper part is the horizontal plane passing through the lower end of the thyroepiglottic ligament; its lower part is the horizontal plane passing through 10 millimeters below the glottic plane (this plane coincides with the lower boundary of the glottic area); on the side, the AC is located between two sagittal planes, which pass through the medial ends of the ventricular folds on both sides, and the anterior quarter of the bilateral vocal cords is located between these two planes (10). Poor exposure of the AC, limited safe resection margins, and invasion of the thyroid cartilage may be the main disadvantages of TLM. There are also challenges in achieving comprehensive endoscopic visualization of the AC during the operation, and the positive rate of surgical margins is significantly higher than that of those without involvement (25). Hans et al. (28) reported 148 cases of early glottic cancer, among which 65.9%, 57.4%, and 4.2% of the cases required external counterpressure, partial vestibulectomy, or total vestibulectomy, respectively. 24.8% of the cases required resection of the petiole of the epiglottis. Compared with patients with T1a stage and Tis stage squamous cell carcinoma, patients with T2 stage and T1b stage had a higher proportion of undergoing resection of the petiole of the epiglottis and vestibulectomy. Based on our experience, a complete set of laryngoscopes, combined with the external counterpressure method and vestibulectomy or resection of the petiole of the epiglottis, can enable most AC lesions to be treated. Eker et al. (27) evaluated the interplate angle of the thyroid cartilage by preoperative laryngeal and pharyngeal CT and found that a small angle would lead to poor exposure and increase the risk of recurrence. The median interplate angle of the thyroid cartilage in the non-recurrence group was 71.0, and that in the recurrence group was 64.0. In addition, they evaluated the thickness and vertical length of the AC by preoperative CT and analyzed that the vertical extension ratio (the vertical length of the AC tumor measured in the sagittal plane of CT/the horizontal length at the glottic level) was significantly correlated with disease recurrence (p = 0.001). However, no significant correlation was observed between the thickness of the AC and disease recurrence or overall survival rate. Tumors of AC3 type would progress and spread along the cranial and caudal sides of the AC, but the proportion of spreading to the caudal side was significantly higher than that to the cranial side. There is an independent region called the “0 plane” between the thyroepiglottic ligament and the Broyles ligament, which lacks glands and blood vessels and is therefore considered to be able to effectively prevent tumor infiltration (29). However, glands and blood vessels are continuously present in the glottic and subglottic regions (30). Wu et al. (31) found that when glottic cancer spread to the supraglottic region, 14.8% spread through the AC, and when it spread to the subglottic region, 71.4% spread through the AC. Among the groups with and without vocal cord muscle involvement in the AC area, the incidence of middle layer of thyroid cartilage involvement was 0.0% (0/18) and 53.8% (8/13), respectively. Among the AC2 patients, 6/9 had vocal cord muscle involvement at the AC, and among the AC3 patients, 7/7 had vocal cord muscle involvement at the AC. Given the high probability of middle layer of thyroid cartilage involvement in AC3 type cases, it is recommended not to use TLM. Rucci et al. (32) reported 13 AC2 T1b patients, among which 10 had recurrence, and they recommended open partial laryngectomy for AC2 patients. Marchi et al. (33) note that depth of invasion has been recognized as a critical oncological concept, yet the TNM staging system for laryngeal carcinoma has not incorporated this parameter. Piazza et al. (34) reclassified glottic laryngeal carcinomas based on depth of invasion. Analyzing 410 cases of pT1-pT3 glottic cancer, they found that tumors exhibiting vertical invasion through the AC (with or without pre-epiglottic space involvement) had significantly poorer local control rates compared to superficial localized and superficial diffuse tumors (2-year local control: 66.7% vs. 71.9 – 100%). This pattern of invasion was associated with a significantly increased risk of local recurrence [Hazard Ratio (HR) = 13.3]. Transglottic tumors pose greater challenges for endoscopic resection than horizontally growing ones.

The oncologic outcomes for AC3 are significantly worse compared to AC0 to AC2. Summarizing the reasons requires consideration of the higher rate of thyroid cartilage invasion in AC3 and the anatomically constrained safe resection margins for vertical deep tumor invasion. We believe that for most patients with AC involvement, TLM is still a safe and minimally invasive treatment option. Preoperative evaluation of the tumor growth pattern should be carried out through image-enhanced endoscopy and CT examination. For patients with AC3 stage, TLM should be chosen cautiously. For AC3 laryngeal cancer, open partial laryngectomy serves as the preferred alternative. This approach provides direct exposure of the anterior commissure and thyroid cartilage, enabling en bloc resection of vertically invasive tumors. It allows for clear visualization to ensure negative margins at the cricothyroid membrane and subglottic regions, permits larger resection margins, and eliminates concerns regarding potential invasion of the perichondrium or thyroid cartilage itself.

Compared to previous studies, our cohort includes 25 cases of AC3, but the sample size remains relatively small. This may affect the statistical robustness of conclusions regarding AC-III’s impact on oncologic outcomes. Anatomical challenges specific to AC3 (such as thyroid cartilage invasion and vertical spread) require validation in larger cohorts.

## Conclusion

TLM shows good oncological efficacy in the treatment of T1a and T1b glottic cancer. Our study provides evidence that it is not the T stage but the severe involvement of the AC that affects the prognosis of T1a and T1b. For patients with severe AC3 involvement, TLM should be chosen cautiously; preoperative assessment combining image-enhanced endoscopy and CT is essential to evaluate tumor growth patterns, and open partial laryngectomy may serve as an alternative option.

## Data Availability

The original contributions presented in the study are included in the article/Supplementary Material. Further inquiries can be directed to the corresponding author.
